# Commentary: Fertility-enhancing effect of oil-based contrast agents during hysterosalpingography and the variation of this effect within a 3-year follow-up period in infertile patients

**DOI:** 10.3389/fmed.2023.1287896

**Published:** 2024-01-17

**Authors:** Nienke van Welie, Rik van Eekelen, Kim Dreyer, Velja Mijatovic, Ben Willem Mol

**Affiliations:** ^1^Department of Reproductive Medicine, Amsterdam UMC, Vrije Universiteit Amsterdam, Amsterdam, Netherlands; ^2^Amsterdam Reproduction and Development Research Institute, Amsterdam, Netherlands; ^3^Centre for Reproductive Medicine, Amsterdam UMC, University of Amsterdam, Amsterdam, Netherlands; ^4^Department of Obstetrics and Gynaecology, Monash University, Clayton, VIC, Australia; ^5^Aberdeen Centre for Women's Health Research, University of Aberdeen, King's College, Aberdeen, United Kingdom

**Keywords:** HSG, fertility, therapeutic effect, oil-based contrast, water-based contrast, follow-up

You recently published an article by Lu et al. (1) on the 3-year follow-up period in infertile patients undergoing hysterosalpingography with oil- or water based contrast agents (1). There are a lot of resemblances with an article previously published by our study group (2). While Lu et al. (1) refer to our primary studies (3, 4), they do not refer to our paper with a similar structure. Is there a reason for that? We would like to discuss some other aspects of the paper with the authors.

First, the Kaplan-Meier curve produced by Lu et al. (1) indicate that the difference in pregnancy rates between the two groups is growing over 3 years. This finding is in complete contrast with ours and other studies have shown the treatment effect to occur in the first 4 months and remains stable thereafter (3, 4). Remarkable is also that the Kaplan-Meier curve of Lu et al. (1) has exactly equal steps in both groups. Furthermore, the authors produce data on pregnancies per month and pregnancies per physiological cycle [Tables 2, 3 of Lu et al. (1)]. By our understanding, the pregnancy rates per physiological cycle should be higher, and we therefore are surprised that Lu et al. (1) show the opposite.

Second, Lu et al. (1) report “a spontaneous pregnancy rate of 79.0% in the oil-based group and 70.2% in the water-based group within a 3-year follow-up after HSG.” In contrast, our study (5) had only 40% of the pregnancies (so 30% of the total population) pregnant after natural conception. The 70–79% spontaneous pregnancy rate in an infertile population that is trying to conceive for 2 years as reported by Lu et al. (1) is in our opinion not possible.

Third, Lu et al. (1) report a median age of 27 vs. 29 years in Table 1. While the medians do not allow reproduction of the *P*-values, it is again remarkable that this difference in two groups of 500 is not statistically significant.

Fourth, in Supplementary Table 2 Lu et al. (1) report on other factors that influence fertility. The age is reported to be not statistically significant and if anything older age (HR 1.003) increases fertility. This is also completely in contrast with our understanding of basic biology. Cesarean section is reported to not to affect fertility (HR 0.956) but of course an earlier baby is a beneficial factor for future fertility. Similar for history of tubal pregnancy. All extremely unlikely.

Fifth, Lu et al. (1) report that they collected data from January to June 2018 (that must have been prospective). There are no missing data in a cohort of a 1,000 women. We find it difficult to believe that a sample of 1,000 women were included within 6 months' time in a single hospital, not resulting in any missing data after 3 years of follow-up.

Furthermore, how was the decision made for oil or water; if this was not randomized, how does it end in 500 vs. 500? It is extremely unlikely to happen in the way Lu et al. (1) describe this. They report no information regarding sample size, allocation and blinding, trial registry and they did not publish any study protocol.

To summarize, the data presented in the article of Lu et al. (1) raise many questions. It would be great if Lu et al. (1) could answer our questions and provide the raw data of their study.

## Author contributions

NW: Writing – original draft, Writing – review & editing. RE: Writing – review & editing. KD: Writing – review & editing. VM: Writing – original draft, Writing – review & editing. BM: Writing – original draft, Writing – review & editing.
